# MiR-9-5p Inhibits the MMP^+^-Induced Neuron Apoptosis through Regulating SCRIB/*β*-Catenin Signaling in Parkinson's Disease

**DOI:** 10.1155/2022/9173514

**Published:** 2022-04-25

**Authors:** Zhenyong Xiao, Zhenxing Yan, Xiang Sun, Zhiyuan Zhu, Baoyan Wang, Mengqi Gao, Fengfei Lu, Jian Liu, Zhitao Zong, Hongbo Zhang, Yanwu Guo

**Affiliations:** ^1^Neurosurgery Center, Department of Functional Neurosurgery, The National Key Clinical Specialty, The Engineering Technology Research Center of Education Ministry of China on Diagnosis and Treatment of Cerebrovascular Disease, Guangdong Provincial Key Laboratory on Brain Function Repair and Regeneration, The Neurosurgery Institute of Guangdong Province, Zhujiang Hospital, Southern Medical University, Guangzhou 510282, China; ^2^Department of Neurosurgery, The Fourth Affiliated Hospital of Guangxi Medical University, Liuzhou 545005, Guangxi, China; ^3^Department of Neurology, Zhujiang Hospital, Southern Medical University, Guangzhou 510282, China; ^4^Department of Neurosurgery, Jiujiang Hospital of Traditional Chinese Medicine, Jiujiang, Jiangxi 332005, China; ^5^Department of Neurosurgery, The Second Affiliated Hospital of Nanchang University, Nanchang, 330006, China

## Abstract

The pathogenesis of Parkinson's disease remains unclear that there is no cure for Parkinson's disease yet. The abnormal expressions of certain miRNA are closely related to the occurrence and progression of Parkinson's disease. Here, we demonstrate that miR-9-5p inhibits the dopaminergic neuron apoptosis via the regulation of *β*-catenin signaling which directly targets SCRIB, a tumor suppressor gene. Besides, miR-9-5p improved the motor function of mice with Parkinson's disease. The results of this study suggest that miR-9-5p might be a potential therapeutic target against Parkinson's disease.

## 1. Introduction

Parkinson's disease (PD) is the second most common neurodegenerative disease worldwide [1]. The main clinical manifestations of the patient were progressive aggravation of myotonia, bradykinesia, and static tremor [2]. Patients suffer both in physical and in mental. Mitochondrial dysfunction, oxidative stress, neuroinflammation, and excitatory toxicity were considered as the pathogenesis of Parkinson's disease [3, 4]. All of the above pathological processes would lead to dopaminergic neurons dysfunction [1, 3, 4]. Therefore, the repair of damaged dopaminergic neurons and the restoration of dopamine regulation are the fundamental goals in Parkinson's disease therapy [5–14].

MicroRNA (miRNA, miR) regulates the gene expression. The aberrant expression of miRNA involves in the occurrence and development of various diseases, including Parkinson's disease. Due to its easy access and stable expression in body fluids, miRNAs are presumed as potential biomarkers for the diagnosis of PD in early stage and for the monitoring of PD development [5, 6]. Data has showed that the expression of hsa-miR-221-3p, hsa-miR-214-3p, hsa-miR-29c-3p, and miR-124 family is meaningful for the diagnosis of PD [7–10]. Meanwhile, some scholars believe that miRNA has great potential in treating PD [9–14]. The abnormal expression of miR-9-5p in neurodegenerative diseases would sabotage its neuroprotective effect [15, 16]. MiR-9 is closely related to the progress of Alzheimer's disease, directly targeting BACE1, PSEN1, SIRT1, and CAMKK2 [17]. Upregulation of dopaminergic neurons miR-9-5p was detected in PD patients, induced by pluripotent stem cells (iPSCs) [18]. A latest system analysis revealed that the protective effect of miR-9-5p on neurons was contributed to a negative feedback regulatory response [19]. The expression of miR-9-5p in Parkinson's disease may be dynamic, and its underlying mechanisms will be explored further.

## 2. Methods

### 2.1. Cell Culture

Mouse dopaminergic neuron MN9D was purchased from American Type Culture Collection (ATCC) and cultured in Dulbecco's Modified Eagle Medium (DMEM) with 10% fetal bovine serum (FBS) and 1% penicillin-streptomycin (Sigma-Aldrich, USA), under the condition of 37°C and 5% CO_2_. MN9D was treated with 1 mM MMP^+^ (Sigma, USA) for 24 h as a PD model in vitro.

### 2.2. Animals

C57BL/6 mice were used in the present study, and each group contains 5 mice. The PD model was conducted by 5days intraperitoneal injected MPTP (30 mg/kg) and following 10 days intravenous injected agomir (micrON mmu-miR-9-5p and control miRNA, RiboBio, China, 100 nmol/kg). Neurological function of the mice was assessed at day 0, day 1, day 2, day 3, day 7, day 14, and day 21 when MPTP treatment finished. All the mice were killed at day 21 to harvest the midbrain. This study was carried out in strict accordance with the recommendations in the Guide for the Care and Use of Laboratory Animals of the National Institutes of Health. The protocol was approved by the Committee on the Ethics of Animal Experiments of the Southern Medical University.

### 2.3. Gene Transfection

Following the manufacturers' instruction, lipofectamine 3000 (Thermo Fisher, USA) was used for gene transfection. The concentration of mmu-miR-9-5p mimics and negative control miR (RiboBio, China) was 25 nM. miRNAs were mixed with lipofectamine 3000 in serum-free DMEM for 20 min at room temperature; afterwards, the mixture was added into culture dishes. The culture medium was replaced 4 hours later, and the cells were incubated for another 48 h-72 h before harvest. DNA transfection was conducted with the same protocol.

### 2.4. Western Blot

Cell and tissue lysates were prepared with sonication in modified RIPA buffer (Solarbio, China) adding protease inhibitor (Solarbio, China). BCA protein assay kit (Solarbio, China) was used for protein quantification. Protein stripes were visualized with ECL reagents (Solarbio, China). The following primary and secondary antibodies were used in the study: anti-GAPDH (abcam, USA), anti-cleaved caspase 3 (affbiotech, China), anti-Bcl-2(abcam, USA), anti-SCRIB (proteintech, China), HRP-goat anti-rabbit (proteintech, China), and HRP-goat anti-mouse (proteintech, China).

### 2.5. Real-Time PCR

Total RNAs of tissues and cells were extracted using TRIzol Reagent (Invitrogen, USA). Reverse transcription kit (TaKaRa, Japan) and Sybr Pre-mix EX Taq II (Takara, Japan) were performed in mRNA reverse transcription and cDNA amplification, respectively. All in-One miRNA qRT-PCR Detection Kit (GeneCopoeia, Rockville, MD, USA) was used to detect the miRNA expression. The sequence of the primers are listed below: mmu-miR-9-5p forward primer: 5′CCGGTCTTTGGTTATCTAGCTG3′; reverse primer: 5′CTCAACTGGTGTCGTGGAGTC3′; U6 forward primer: 5′CTCGCTTCGGCAGCACAT 3′; reverse primer: 5′AAATATGGAACGCTTCACGA3′. SCRIB forward primer: 5′AACGCTTCACGAATTTGCGT 3′; reverse primer: 5′TCACCAACTCGGACTCCAGC3′. The relative expression of miRNA and mRNA was calculated using the 2^−*ΔΔ*CT^ method.

### 2.6. Flow Cytometry

Cell apoptosis was evaluated by flow cytometry, using FITC Annexin-V (Becton Dickinson). The washed cells were resuspended in binding buffer at a final concentration of 1 × 10^6^/ml. According to the manufactures' instruction, AV-FITC and/or PI were added into the tube and incubated at room temperature for 15 min in dark. Then, samples were detected in an hour.

### 2.7. Luciferase Reporter Assay

Putative binding site between mmu-miR-9-5p and SCRIB was predicted by the miRNA database (http://www.targetscan.org). The mmu-miR-9-5p sequence binding to the 3′UTR of SCRIB, either wildtype or mutant, was cloned into the pMIRREPORT vector (Ambion, USA). MN9D cells were cultured in 24-well plates and transfected with 0.1 *μ*g of luciferase reporter vectors contained mmu-miR-9-5p mimics or control miRNA. Renilla luciferase-expressing vector (pRL-TK, Promega, USA) was cotransfected for normalization. Cells were harvested after 48 h transfection. According to the manufacturer's instruction, Firefly and Renilla luciferase activities were detected using the Dual-Luciferase Reporter Assay System (Promega).

### 2.8. immunofluorescence

After anesthetized with overdosed barbiturate, mice were transcardially injected with 4% paraformaldehyde, and brain tissues were handled for immunofluorescence as previously described [20]. Antibodies were listed below: anti-TH (servicebio, China), anti-cleaved-caspase 3 (proteintech, China), FITC, and CY3 antibodies (APSEN, China).

### 2.9. Statistical Analysis

Data were expressed as mean ± standard error. Statistical analysis was performed with SPSS 20.0 software. Differences between means were assessed by Student's *t*-test for normal distribution data or Mann–Whitney *U* test for nonnormal distribution data. In multiple comparisons, one-way analysis of variance (ANOVA) was adopted. A value of *P* < 0.05 was considered statistically significant.

## 3. Results

### 3.1. MPTP Induces Apoptosis in Dopaminergic Neurons

**Figure 1:**
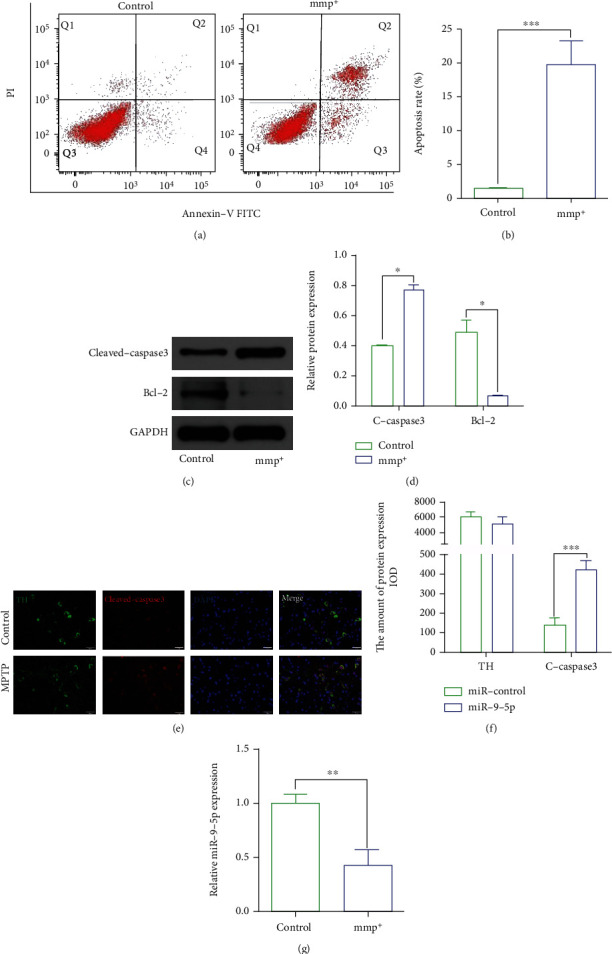
MPTP induced the apoptosis of dopaminergic neurons MN9D. (a, b) The apoptosis rate of MN9D cells in the mmp^+^ group was higher than that in the control group (*P* < 0.05). (c, d) The expression of cleaved-caspase 3 was higher in the mmp^+^ group than in the control group, and the expression of bcl-2 was lower (*P* < 0.05). (e, f) The expression of cleaved-caspase 3 was higher in PD mice (*P* < 0.001). The expression of TH was lower (*P* > 0.05). (g) The expression of mmu-miR-9-5p was decreased in the mmp^+^ group than in the control group (*P* < 0.05). Scale bar: 100 *μ*m.

Abnormal apoptotic signaling is involved in the progression of neurodegenerative diseases, including PD [21]. In this study, apoptosis was increased in MPTP-treated MN9D cells. Flow cytometry showed that the apoptosis rate in MN9D cells treated with MMP^+^ was significantly higher than that of the control group (Figures 1(a) and 1(b)). Meanwhile, the protein expression of cleaved-caspase 3 was higher in the later than the former, and the protein expression of bcl-2 was the opposite (Figures 1(c) and 1(d)). In vivo, cleaved-caspase 3 was upregulated in nigrostriatal system in MPTP-treated mice (Figures 1(e) and 1(f)). To investigate whether miR-9-5p is involved in PD, we examined the level of mmu-miR-9-5p. Results showed that the expression of mmu-miR-9-5p was reduced in MMP^+^-treated MN9D (Figure 1(g)).

### 3.2. mmu-miR-9-5p Alleviates the MPTP-Induced Apoptosis in Dopaminergic Neurons

**Figure 2:**
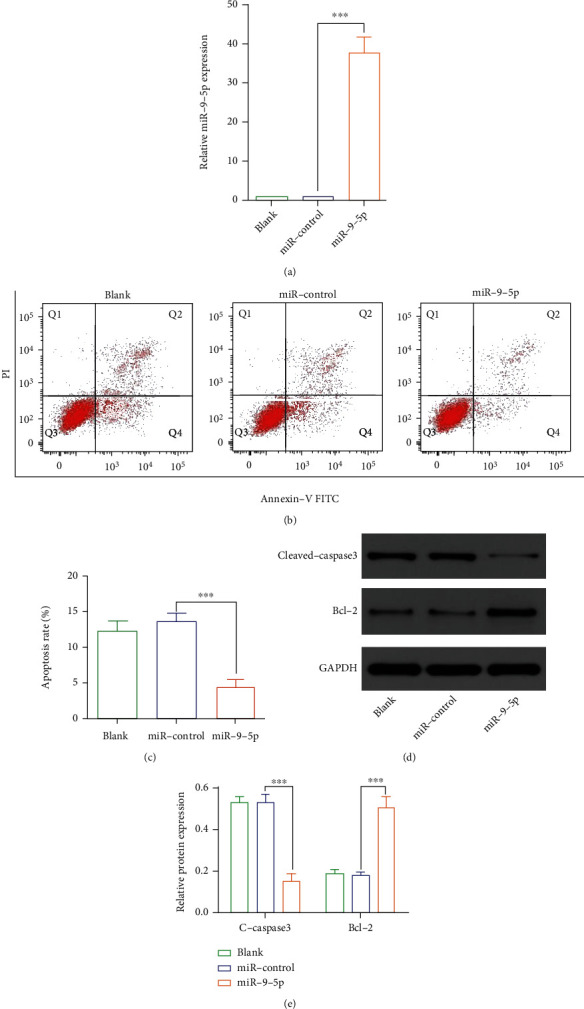
mmu-miR-9-5p reversed the MPTP-induced apoptosis of dopaminergic neurons. (a) The expression of mmu-miR-9-5p was upregulated after transfected with mmu-miR-9-5p mimics (*P* < 0.05). (b, c) The apoptosis rate of MN9D cells was reduced in the mmu-miR-9-5p group than in the miR-control group (*P* < 0.05). (d, e) Western blot showed cleaved-caspase 3 was downregulated, and bcl-2 was upregulated (*P* < 0.05). The blank group stands for untreated normal cells, the miR-control group was transfected with negative control miRNAs, and the miR-9-5p group was transfected with mmu-miR-9-5p mimics.

We tried to restore the expression of mmu-miR-9-5p in the PD cell model. With gene transfection, we successfully enhanced the expression of mmu-miR-9-5p in MN9D cells (Figure 2(a). Then, we evaluated the trend of apoptosis. The apoptosis rate of mmu-miR-9-5p treated cells was decreased (Figures 2(b) and 2(c)). Western blot showed that the expression of cleaved-caspase 3 was reduced while the expression of bcl-2 was upregulated in mmu-miR-9-5p treated cells (Figures 2(d) and 2(e)).

### 3.3. mmu-miR-9-5p Involves in Multiple Signaling Pathways of Neuronal Apoptosis

**Figure 3:**
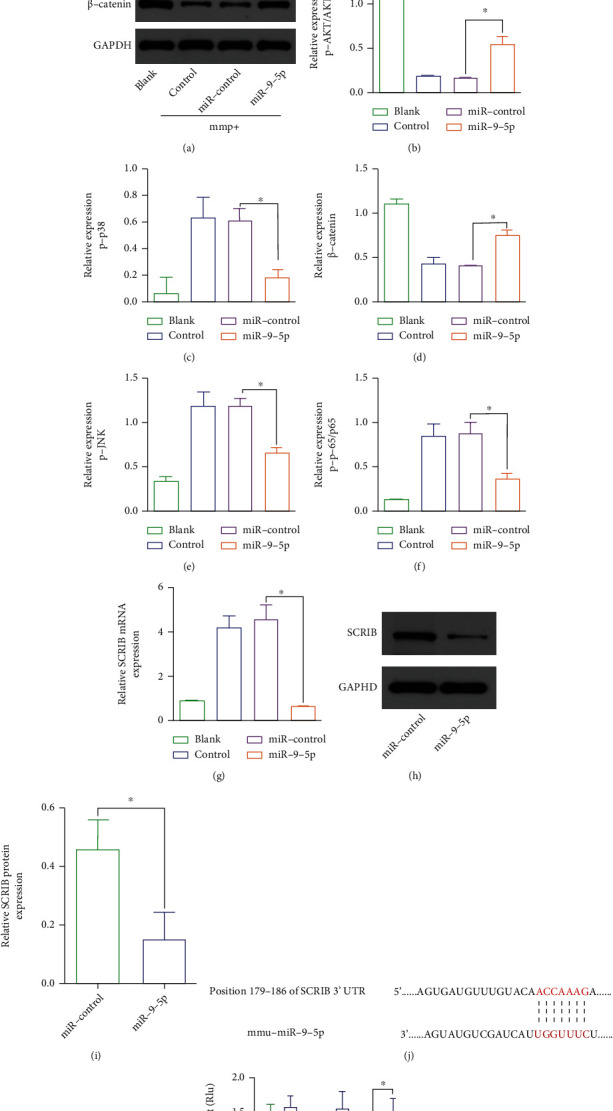
mmu-miR-9-5p regulates the SCRIB/*β*-catenin signaling pathway by directly targeting SCRIB. Western blot was used to detect possible signals for apoptosis. (a, b, d) p-AKT and *β*-catenin were upregulated (*P* < 0.05). (a, c, e, f) p-p38, p-JNK, and p-p65 were downregulated (*P* < 0.05). (g) The expression of SCRIB mRNA was upregulated in the mmp^+^ group than the control group (*P* < 0.05) and was inhibit by mmu-miR-9-5p (*P* < 0.05) (h, i) The expression of SCRIB was downregulated in the mmu-miR-9-5p group (*P* < 0.05) detected by Western blot. (j) Predicted binding sites of mmu-miR-9-5p and SCRIB. (k) Luciferase reporter assay suggests SCRIB is the direct binding to mmu-miR-9-5p (*P* < 0.05).

To clarify how mmu-miR-9-5p inhibits MMP^+^-induced neuronal apoptosis, we examined signaling pathways associated with neuronal apoptosis. Results found that mmu-miR-9-5p improved the activity of *β*-catenin and Akt signaling which was previously suppressed by MMP^+^. On the contrast, the p-38/JNK signal was inhibited (Figures 3(a)–3(e)). However, the p65 signaling pathway was suppressed, which promotes cell survival. (Figures 3(a) and 3(f)).

### 3.4. mmu-miR-9-5p Regulates the *β*-Catenin Signaling Pathway by Directly Targeting SCRIB

It has been reported that SCRIB directly regulates *β*-catenin activity [22], and database suggested that SCRIB was one of the mmu-miR-9-5p targeted genes (Figure 3(j)). To further explore the relationship between mmu-miR-9-5p and SCRIB, we tested the SCRIB expression. Mmu-miR-9-5p inhibits the mRNA and protein expression of SCRIB that induced by MMP^+^ (Figures 3(g)–3(i)). Luciferase demonstrated that mmu-miR-9-5p directly binds to SCRIB (Figure 3(k)).

### 3.5. SCRIB Inhibits the Protective Effect of mmu-miR-9-5p on Apoptotic Cell

**Figure 4:**
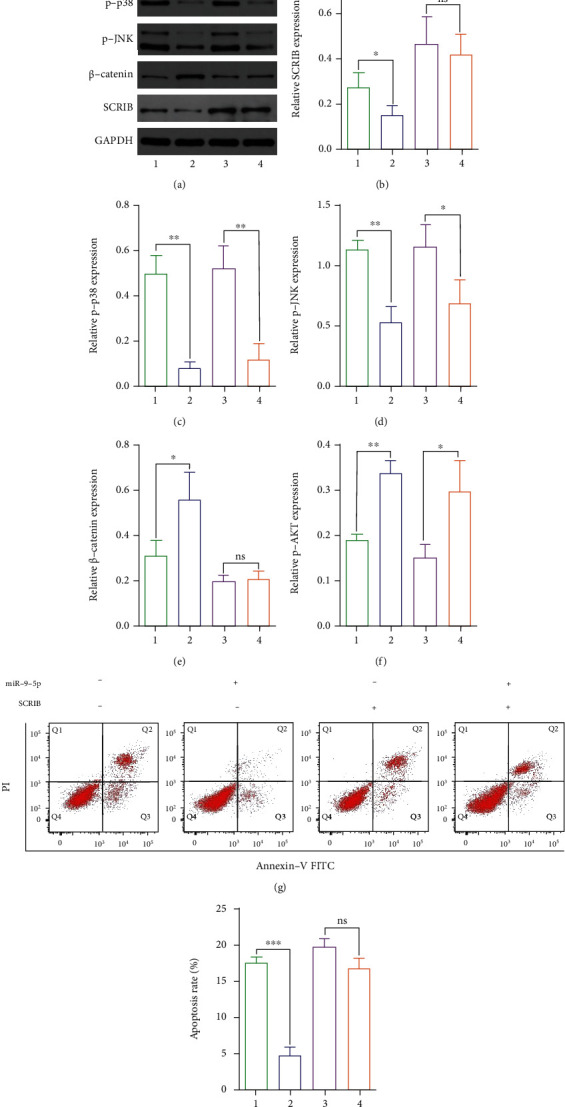
SCRIB inhibits the protective effect of mmu-miR-9-5p on apoptotic cell. Western blot showed that after the SCRIB expression was restored, mmu-miR-9-5p lost its regulation of *β*-catenin (a, b, e), while the expression of p-p38,p-JNK, and p-AKT was not affected (a, c, d, f). (g, h) The apoptosis rate of MN9D cells was also out of control of mmu-miR-9-5p after the SCRIB expression was restored (*P* > 0.05).

To testify whether mmu-miR-9-5p regulates *β*-catenin by SCRIB or not, we restored the expression of SCRIB Figures 4(a) and 4(b)). Consequently, mmu-miR-9-5p lost its control to *β*-catenin (Figures 4(a) and 4(e)) without affecting other signals (Figures 4(a), 4(c), 4(d), and 4(f)). Flow cytometry indicated that apoptosis of PD cells were increased when SCRIB was presented, compared to the mmu-miR-9-5p group only (Figures 4(g) and 4(h)).

### 3.6. mmu-miR-9-5p Improves the Behavior of PD Mice

**Figure 5:**
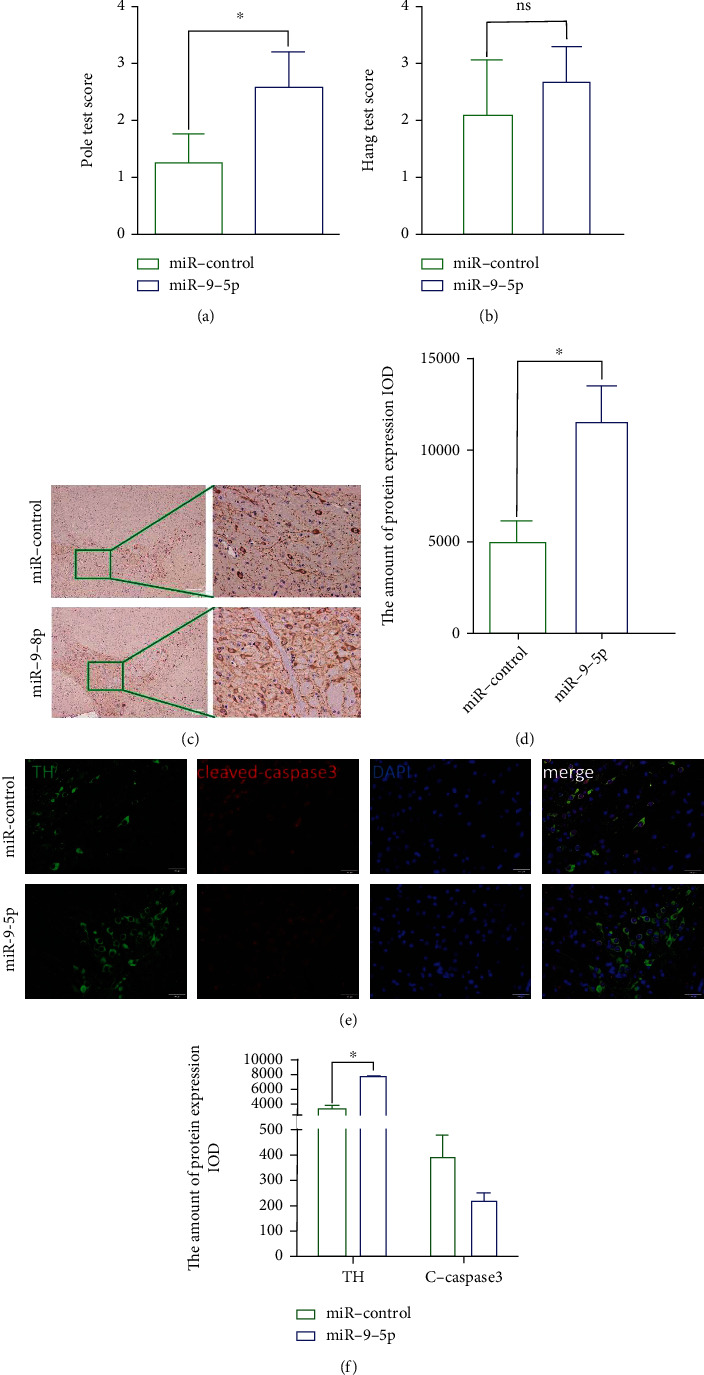
mmu-miR-9-5p improved the behavior of PD mice. Pole test (a) and hang test (b) were used to evaluate the motor function of mice. (c, d) Immunofluorescence showed that TH positive cells were significantly higher than the control group (*P* < 0.05). (e, f) The expression of cleaved-caspase 3 was higher in the mmu-miR-9-5p group (*P* > 0.05). The expression of TH was lower (*P* < 0.05). Scale bar: 100 *μ*m.

To verify the effect of mmu-miR-9-5p in vivo, mmu-miR-9-5p was administered in PD mice model. It seemed that athletic ability of PD mice was improved (Figures 5(a) and 5(b)). Furthermore, brain tissue staining showed more TH-positive cells in the mmu-miR-9-5p group than in the control group (Figures 5(c) and 5(d)). Immunofluorescence results showed that the expression of TH in the mir-9 treated group was higher than that in the untreated group, and there was no statistical significance between the two groups in the cleaved-caspase 3 expression (Figures 5(e) and 5(f)).

## 4. Discussion

The decrease or dysfunction of dopaminergic neurons is the main cause for Parkinson's disease [23]. Apoptosis, a programmed cell death, is an effective way to eliminate the aged or aberrant cells, thus maintain the self-renewal of organs [24]. The abnormality of the apoptosis is one of the pathogenesis of many neurodegenerative diseases, such as Parkinson's disease, and subsequently leads to the loss of dopaminergic neurons in the substantia nigra pars compact [25, 26]. The measurements that promote apoptosis could be used against the progression of Parkinson's disease and to improve the patients' prognosis.

MiR-9-5p is crucial in the development of the nervous system, targeting different mRNAs. It regulates several physiological processes in neural precursor cells, such as proliferation, migration, and differentiation [27]. The role of miR-9-5p in neurodegenerative diseases is complex. Studies have shown that the expression of miR-9-5p is changed with time and lesion site depended in Alzheimer's disease [15, 16, 28–30], which means the roles of miR-9-5p relying on neuron types. The expression of serum miR-9-5p was significantly higher in treated Parkinson's patients than untreated Parkinson's patients and healthy people [31]. MiR-9-5p is upregulated in PD patients' dopaminergic neurons via somatic cell reprogramming and induced pluripotent stem cells' differentiation [18]. Our evidence demonstrated that miR-9-5p protects dopaminergic neuron from apoptosis which induced by MMP^+^. Taken together, the neuroprotective effect of miR-9-5p is undisputed. The upregulation of miR-9-5p in Parkinson's disease might be a self-protective feedback.

Drug-induced *β*-catenin signaling is effectively counteracted the toxicity of dopaminergic neurons, leading to neuroprotection and neurorestoration [32–34]. Our data showed that miR-9-5p activates *β*-catenin signaling which was inhibited by MPTP in dopaminergic neurons. MiR-9-5p has potential to be a therapeutic agent for PD. Besides, studies suggest that *β*-catenin is a vital pathway for dopaminergic neurogenesis. The unusual *β*-catenin pathway may precede and/or accompany PD onset and progression [35]. In MPTP treated mice, cell proliferation in subventricular zone (SVZ), riched in neural stem cells, was significantly inhibited with decrease of *β*-catenin signal [34]. Therefore, miR-9-5p administration may promote the proliferation and differentiation of neural stem cells through activating *β*-catenin signaling and regain the dopaminergic neurons as well.

Studies have shown that SCRIB bind to *β*-catenin form stable complexes, promoting *β*-catenin degradation [22]. Our data suggested that SCRIB is the target of miR-9-5p, and the regulation of miR-9-5p on *β*-catenin is not direct but realized by SCRIB.

## 5. Conclusion

MiR-9-5p inhibits the apoptosis of dopaminergic neurons in PD and improves the symptoms of Parkinson's disease, involving in a variety of signaling pathways. MiR-9-5p upregulates *β*-catenin signaling pathway by directly targeting SCRIB. In conclusion, miR-9-5p has great potential to be a therapeutic target for Parkinson's disease.

## Data Availability

The article data used to support the findings of this study are included within the article. The mir-9 database is on the http://mirdb.org.

## Additional Points

*Contribution to the field.* Parkinson's disease is a complex challenge of neuroregulatory disorder. There is still no cure for Parkinson's disease, and even the pathogenesis of Parkinson's disease remains unclear. The abnormal expression of miRNA is closely related to the occurrence and progression of Parkinson's disease. Here, we demonstrated that miR-9-5p inhibited the apoptosis of dopaminergic neurons by regulating *β*-catenin signaling, directly targeting SCRIB, which was regard as a tumor suppressor gene. Besides, miR-9-5p improved the motor function of mice with Parkinson's disease. The results of this study suggest a potential therapeutic target for Parkinson's disease.
